# A robust and efficient algorithm for Chinese historical document analysis and recognition

**DOI:** 10.1093/nsr/nwad115

**Published:** 2023-04-25

**Authors:** Chongyu Liu, Cheng Jian, Jiarong Huang, Wentao Yang, Yongxin Shi, Qing Jiang, Lianwen Jin

**Affiliations:** School of Electronic and Information Engineering, South China University of Technology, China; School of Electronic and Information Engineering, South China University of Technology, China; School of Electronic and Information Engineering, South China University of Technology, China; School of Electronic and Information Engineering, South China University of Technology, China; School of Electronic and Information Engineering, South China University of Technology, China; School of Electronic and Information Engineering, South China University of Technology, China; School of Electronic and Information Engineering, South China University of Technology, China; SCUT-Zhuhai Institute of Modern Industrial Innovation, China

## Abstract

This paper presents a novel and efficient algorithm for Chinese historical document understanding, incorporating three key components: a multi-oriented text detector, a dual-path learning-based text recognizer, and a heuristic-based reading order predictor.

## PROBLEM

Historical documents, which contain valuable information on historical knowledge and literary arts, are important mediums for the inheritance of human civilization. Therefore, the preservation of historical documents is considered to be an urgent research topic. One efficient way is to develop a document digitization system, which requires accurate historical document recognition and understanding. With the rapid development of optical character recognition techniques, historical document understanding has made great progress [1,2] and several benchmarks have been established [3–5]. Nevertheless, real-world scenarios present several challenging artifacts [6], such as substantial variations in page layouts, image degradation, as well as diversity in text fonts and scales, which have seldom been taken into account in the current benchmarks. Though the relative methods have reported promising performance, they do not perform very well in the aforementioned situations. Aiming at inviting the community to work toward good solutions to address the aforementioned challenges, the 2022 Greater Bay Area (Huangpu) International Algorithm Case Competition for Chinese Historical Document Recognition and Analysis Challenge was organized with a new challenging Chinese Historical Document Analysis Challenge (CHDAC) benchmark. To more comprehensively evaluate the performance and limitations of historical document understanding systems, the following two tasks were considered in the competition.

- **End-to-end text detection and recognition.** This task can be separated into text detection and text recognition. The objective of the former is to detect the body text of the documents, ignoring text such as volume numbers and legends. The latter is to recognize each character in each cropped text image patch. Accurate text detection and recognition is the cornerstone of a historical document digitization system.
- **Reading order prediction.** The objective of this task is to arrange all of the detected text into the correct reading order. The correct reading order can accurately sort out passages and contents of the text, which is essential for understanding the structure of historical documents.

## CHDAC BENCHMARK

A new benchmark named CHDAC was established for this competition, which consists of 8000 document images collected from various Chinese historical books, covering genealogy, medical books, Buddhist scriptures and local chronicles. Of these, 4000 images were provided by South China University of Technology, and the remaining 4000 images were provided by participants from Baidu Inc. and the Institute of Automation of the Chinese Academy of Sciences. The dataset was randomly divided into training, validation and test sets of 4000, 1000 and 3000 images, respectively. The CHDAC dataset contains numerous challenging scenarios, including complex layouts, image degradation, diversity of text fonts and scales, text distortion. The bounding boxes and their corresponding text content of the body text are annotated and rearranged in the correct reading order.

## ALGORITHM

**Figure 1.**
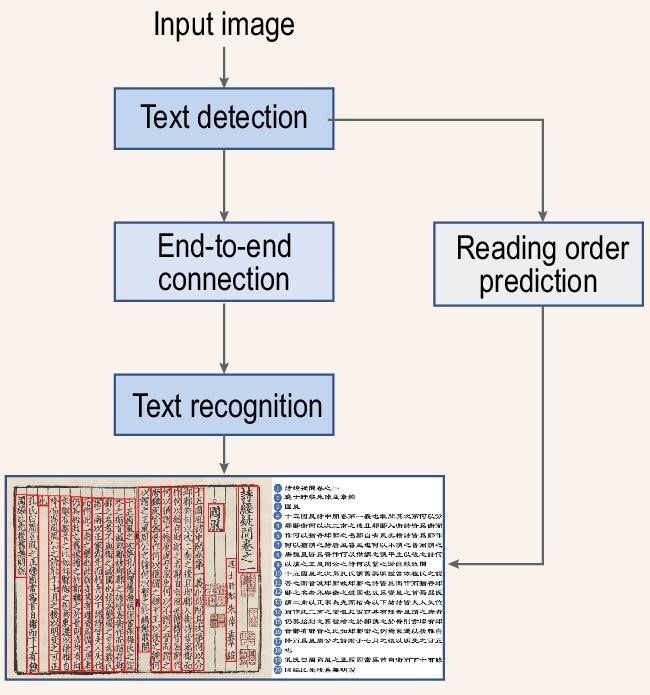
The pipeline of the proposed algorithm.

Our algorithm won the first prize out of 44 participants/teams that registered in the competition. The overall pipeline of our method is shown in Fig. 1. We address the two tasks above with four components: (a) a novel text detection method with multi-oriented region proposal network (RPN) and Iterative Region of Interest (RoI) head; (b) a text recognition method with dual-path learning mechanism; (c) an effective end-to-end connection mechanism between detection and recognition; (d) a heuristic-based approach for reading order prediction.

### Text detection for historical documents

Figure 1 in the online supplementary material shows the pipeline of our proposed detection model. It follows the framework of Mask R-CNN, but differs at its core by using HorNet-tiny [7] with the feature pyramid network backbone, incorporating a multi-oriented RPN, and leveraging an iterative RoI head to greatly improve the performance on the challenging task of text detection from complex historical documents.

Historical document images frequently contain oriented and dense texts. For detecting these oriented objects, a previous study [8] has proposed predicting relative gliding offset on each side of the horizontal bounding boxes. However, it still performs oriented bounding box regression on horizontal proposals from standard RPN, which will result in the detected proposals or bounding boxes for these texts being easily suppressed by non-maximum suppression. To tackle this problem, we utilize the multi-oriented RPN [9] to generate rotated region proposals. Multi-oriented RPN, in contrast to the conventional RPN with {*x, y, w, h*}, integrates two extra parameters, denoted Δα and Δβ, for generating candidate rotated proposals that can effectively deal with the multi-directional distribution of text lines in historical documents. Here Δα and Δβ denote the offsets to the midpoints of upper and right boundaries. The proposal representation *R* = {ϵ_1_, ϵ_2_, ϵ_3_, ϵ_4_} is shown on the right of Fig. 1 in the online supplementary material, which can be formulated as


(1a) \begin{eqnarray*} \varepsilon _{1} \!=\! (x, y - h/2) \!+\! (\Delta \alpha , 0), \end{eqnarray*}

(1b) \begin{eqnarray*} \varepsilon _{2} \!=\! (x + w/2, y) + (0, \Delta \beta ), \end{eqnarray*}

(1c) \begin{eqnarray*} \varepsilon _{3} \!=\! (x, y + h/2) + (-\Delta \alpha , 0), \end{eqnarray*}

(1d) \begin{eqnarray*} \varepsilon _{4} = (x - w/2, y) + (0, -\Delta \beta ). \end{eqnarray*}

After the rotated region proposals are generated, we design a novel iterative RoI head with an iterative regression mechanism. The iterative regression mechanism feeds the RoI features into the same box head and mask head three times to perform iterative prediction with the Intersection over Union (IoU) thresholds of {0.5, 0.6, 0.7}. Compared to the original cascade structure [10], the proposed iterative structure shares the same parameters for each stage, allowing it to achieve comparable performance with fewer model parameters. In addition, to alleviate the adverse effects of text scale variation, rotated RoI align is applied to feature maps in different scales. Finally, we take the prediction of mask head as the detected results.

### Text recognition for historical documents

For text recognition, we propose a dual-path learning model, which consists of a segmentation branch [11] and a Connectionist Temporal Classification (CTC) branch [12]. As shown in Fig. 2 in the online supplementary material, text images are first fed into the ResNet-18 backbone to obtain feature maps and then bifurcated into these two different recognition branches. The segmentation branch can be divided into three individual heads for predicting character location *p_loc_*, character bounding boxes *p_bbox_* and character classification *p_cls_*, separately. This character-based scheme is more robust for long text recognition and alleviates the defects from image degradation or destruction. The CTC branch performs an implicit segmentation-based image-to-sequence translation, which is served as an auxiliary recognizer to predict the text sequence *p_ctc_*. Meanwhile, it can also incorporate semantic information to further improve the model performance. Therefore, our dual-path learning mechanism can integrate both explicit and implicit segmentation-based methods into a unified text recognizer. By leveraging the complementary nature of these approaches, our proposed mechanism yields better recognition results. In the training phase, as CHDAC lacks the character-level annotation, we propose a weakly supervised training paradigm based on a pseudo-label update for the optimization of the segmentation branch. In the inference phase, we only use the CTC branch to decode the final results, which can effectively speed up the inference process.

As the inference time and parameter storage of the model are also considered important metrics, we adopt the FP16 quantization scheme with Torch-TensorRT for speed acceleration and model compression without sacrificing performance.

### End-to-end connection

As our detection output is text region masks, and in order to crop the correct text images for the subsequent recognition model, we propose a cropping method named detection point correction thin plate spline (DPC TPS) to find the exact start and end points for the detected text regions. DPC TPS corrects the detection points at the beginning and end of each mask by drawing an extension line to cover the whole mask region, and then four vertices are extracted from the end of the extension line. The cropped regions by these four vertices can therefore maintain the entire text regions and eliminate distracting areas. TPS transformation is then applied to produce the rectified text image for recognition. Compared to directly extracting regions based on the detection results, our method can obtain more precise cropped text instances.

### Reading order prediction

The reading order of historical documents is governed by a strong prior rule, which is to read from right to left and from top to bottom. Based on this observation, we propose a heuristic-based approach to parse the reading order, which consists of the following steps and is detailed in Algorithm 1 below. (1) The detected text boxes are rotated to make them horizontal if the majority of the text boxes have large skew angles. (2) The rotated text boxes are separated into different paragraphs according to their coordinate projection on the *Y* axis, and then these paragraphs can be parsed in a top-to-bottom order. (3) The text boxes in each paragraph are aggregated into distinct columns according to their adjacency relationship. In this way, different columns can be parsed in a right-to-left order. (4) Given the text boxes in each distinct column, their internal reading order can be parsed on the rules top to bottom and right to left. With a hierarchical combination from steps 2–4, the reading order of the whole detected text boxes can be parsed.

**Algorithm 1:** Pseudo-code of the reading order prediction

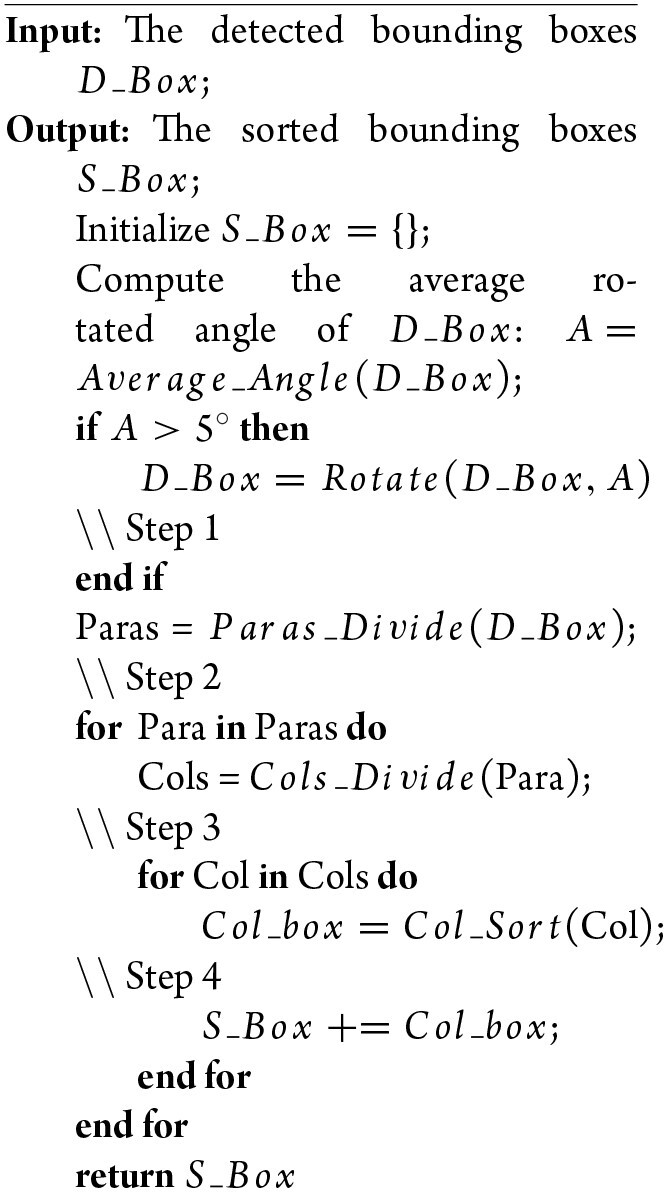

## EVALUATION AND RESULTS

### Evaluation metrics

The evaluation metric for **Task 1** is the normalized edit distance (NED). We calculate the Levenshtein distance between the detected result and the matched ground truth (IoU higher than 0.5 or maximum IoU). The normalized metric *Norm*_1_ equals 1 − *NED*, which is formulated as


(2) \begin{eqnarray*} Norm_{1} &=& 1 - \frac{1}{N}\sum _{i=1}^{N}\\ && \frac{edit\_dist(s_{i1}, s_{i2})}{\max (l_{1}, l_{2})}, \end{eqnarray*}

where *s*_*i*1_ and *s*_*i*2_ denote the predicted text and the matched ground-truth text, respectively, and *l*_1_ and *l*_2_ are their text lengths.

The evaluation metric of **Task 2** is based on the average relative distance (ARD). It measures the relative distance between the common elements in the predicted text orders and the ground truth, which is formulated as


(3) \begin{eqnarray*} && s(e_k, B) = \\ && \,\, \left\lbrace \begin{array}{@{}l@{\quad }l@{}}|k - I(e_k, B)| & \mbox{if }e_k \in B,\\ n & \mbox{otherwise}, \end{array}\right. \end{eqnarray*}

(4) \begin{eqnarray*} \text{ARD}(A, B) = \frac{1}{n} \sum _{e_k\in A} s(e_k, B), \end{eqnarray*}

(5) \begin{eqnarray*} Norm_{2} = \frac{1}{N}\sum _{i=1}^{N}\text{ARD}(A_{i}, B_{i}), \end{eqnarray*}

where *A* and *B* denote the text order of the ground truth and prediction, respectively; *e_k_* is the *k*th text instance in *A* and *I*(*e_k_*, *B*) represents the index of *e_k_* in *B*.

The final scores are obtained by weighting *Norm*_1_ and *Norm*_2_ by 0.8 and 0.2, respectively. In addition, the inference time and storage size of the models are also taken into account for evaluation.

### Results and highlights

Our method achieved 91.43% *Norm*_1_ on the final 3000 testing images for Task 1, which was the best result; and obtained 1.61 *Norm*_2_, ranking in the top three of all submissions for Task 2. Moreover, the inference speed was an average of 505 ms per image and the model parameters took 187 MB in total. Both of them ranked in the top three of all participants/teams. Besides, the floating-point operations per second of our detection model and recognition model are 806.65 G and 57.10 G, respectively. Table 1 in the online supplementary material presents a comparison between our method and the top-five methods in terms of inference time and model size. Our final score (97.95) for the overall performance won the first place, outperforming the second place by 2.05, which proves the effectiveness of our approach.

In our design, we proposed elaborate solutions to the inherent problems of the existing historical documents. For text detection, in addition to utilizing multi-oriented RPN for detecting rotated and irregular dense texts, we incorporated an iterative regression mechanism (IRM) to further improve detection performance. We have conducted experiments to verify the effectiveness of the proposed IRM compared to the original cascade structure [10]; the quantitative results are shown in Table 2 of the online supplementary material. Our baseline model is built on the Mask R-CNN framework, which integrates a ResNet-50 as its backbone and a multi-oriented RPN. Both the cascade structure and our IRM can enhance the performance by approximately 0.4 in terms of the F-measure. Nevertheless, our IRM is considerably more lightweight. As for text recognition, we also designed a dual-path learning scheme to avoid the recognition errors caused by long text corruption, meanwhile preserving contextual semantic information. Additionally, visualization results on various complex situations are shown in Figs 3 and 4 in the online supplementary material to demonstrate the effectiveness of our proposed method.

### Generalization ability

Our proposed model can effectively solve the problem of recognizing historical document images in different complex scenarios, and outperforms existing methods while keeping fast inference speed and small model parameters, which is a solution with good generality and practical application value. To validate the generalization performance of our method, we conducted experiments on other publicly historical document benchmarks, including MTH v2 [4] and ICDAR 2019 HDRC CHINESE [5]. In the online supplementary material, the quantitative results for text detection are given in Table 3, while the text recognition results are presented in Table 4. The experiments demonstrate that our method outperforms previous methods on these two datasets.

## FUTURE RESEARCH

Although our method achieves promising performance, there still exist many challenges in this field, such as the long-tailed distribution over the character categories, the generalization to complex, diverse layouts and text styles, the recognition of multi-lingual historical documents. Therefore, various valid research topics remain unsolved and merit further investigation in the future, which are summarized as follows.

- **Zero-shot/few-shot learning for large-scale historical document understanding.** Because of the large size of character categories, diverse writing styles and structure similarity between different characters, there exists a notorious long-tailed distribution issue in historical document recognition. Furthermore, it is almost impossible to collect enough labeled training samples of each category. Therefore, zero-shot/few-shot learning methods deserve more in-depth study.
- **Self-supervised pre-training for historical documents.** Unlabeled historical document data are easy to obtain and could be used for improving the performance of data-driven recognition algorithms. In order to incorporate these free data into the data-driven pipeline, self-supervised learning is a promising emerging technology that is worth investigating.
- **Construct a global platform for automatic information processing of historical documents.** A unified and general platform for historical document understanding would definitely help the preservation and digitization of historical documents, which is still a worldwide problem. However, there is an important challenge that prevents us from such a platform, which is how to deal with the multi-lingual text (Chinese, English and Arabic) in a unified model.

## Supplementary Material

nwad115_Supplemental_File
