# Supplementary material for: A robust and efficient algorithm for Chinese historical document analysis and recognition
Source: Natl Sci Rev. 2023 Apr 25;10(6):nwad115. doi: 10.1093/nsr/nwad115 (PMC10246825; doi:10.1093/nsr/nwad115)
Supplement: nwad115_Supplemental_File [file nwad115_supplemental_file.pdf]

Appendix

1 The pipeline of the proposed text detection and recognition models

In this section, we present the overall architectures of our proposed text detection and recognition models for historical document in Fig. 1 & Fig. 2.

2 More experimental results

2.1 Complexity analysis

The inference time and parameter storage of the model are both considered as important metrics for evaluation in this competition. Table 1 presents a comparison between our method and the Top-5 methods in terms of inference time and model size.

2.2 The effectiveness of iterative regression mechanism (IRM)

We have conducted experiments to verify the effectiveness of the proposed iterative regression mechanism (IRM) compared to original cascade structure [1], the quantitative results are shown in Table 2. Our baseline model is built on Mask R-CNN framework, which integrates a ResNet-50 as backbone and a multi-oriented RPN. Both the cascade structure (CS) and our iterative regression mechanism (IRM) can enhance the performance by approximately 0.4 in terms of F-measure. Nevertheless, our IRM is considerably more lightweight.

2.3 Qualitative results

Fig. 3 & 4 show some visualization results on various complex situations to demonstrate the effectiveness of our proposed method.

2.4 Generalization Ability

To validate the generalization performance of our method, we conduct experiments on other publicly historical document benchmarks, including MTH v2 [2] and ICDAR 2019 HDRC CHINESE [3]. The quantitative results for text detection are given in Table 3 , while the text recognition results are presented in Table 4.

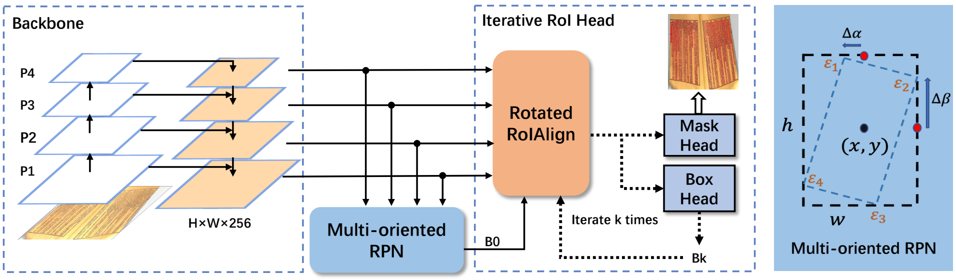

Figure 1: The detection framework of our proposed algorithm.

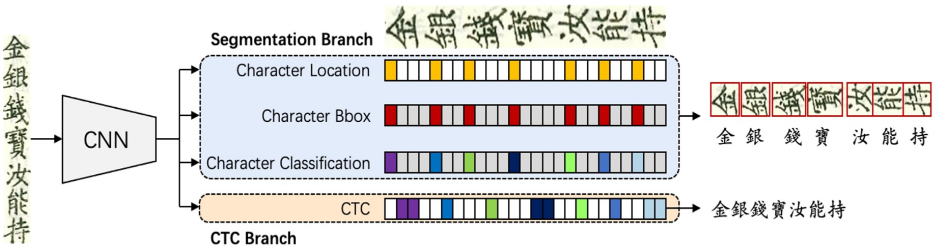

Figure 2: The recognition framework of our proposed algorithm.

Table 1: Quantitative comparisons on inference time and model size of different methods.

Methods	Model Size (Mb)	Infer. Time (ms)
2nd team	173.94	382.52
3rd team	108.71	890.37
4th team	276.11	1218.31
5th team	620.16	2497.93
Ours (w/o FP16)	257.80	–
Ours (w FP16)	187.76	505.61

Table 2: Ablation Study on the proposed iterative regression mechanism. 'CS' denotes Cascade Structure in Cascade R-CNN [1], while 'IRM' represents our iterative regression mechanism.

Methods	Recall	Precision	F-measure	Params (M)	Infer. Time (ms)
baseline	92.82	97.08	94.90	43.99	179.23
w CS [1]	92.73	98.15	95.36	77.04	190.46
w IRM	92.70	98.08	95.31	43.99	189.92

Table 3: Comparison with previous text detection methods on MTH v2 [2] and ICDAR 2019 HDRC CHINESE [3]. Bold indicates SOTA, while Underline indicates second best.

Methods	MTH v2			ICDAR 2019 HDRC CHINESE		
	Recall	Precision	F-measure	Recall	Precision	F-measure
BDN [4]	97.19	98.17	97.68	**94.78**	94.45	94.61
PAN [5]	93.14	97.18	95.12	89.34	93.34	91.30
FCENet [6]	92.82	95.16	93.97	91.11	92.38	91.74
DB++ [7]	94.93	96.20	95.56	91.05	93.10	92.10
Ours	**97.94**	**98.85**	**98.39**	94.19	**95.99**	**95.10**

Table 4: Comparison with previous text recognition methods on MTH v2 [2]. Bold indicates SOTA, while Underline indicates second best.

Methods	AR	CR
Ma et al. [2]	95.52	96.07
Shi et al. [8]	96.94	97.15
Huang et al. [9]	97.42	97.62
Ours	**98.03**	**98.10**

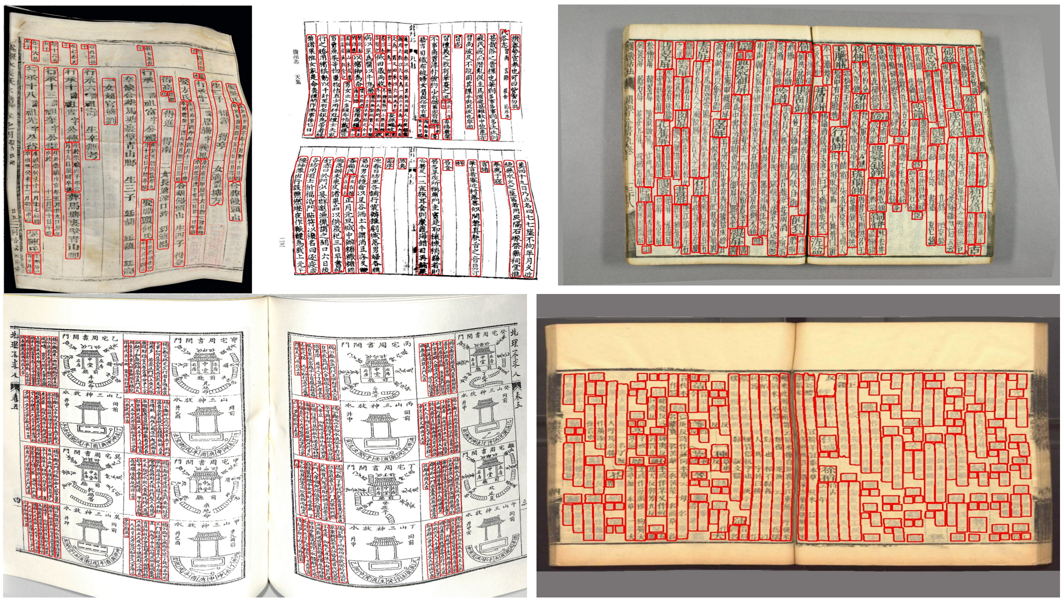

Figure 3: Visualization results on text line detection.

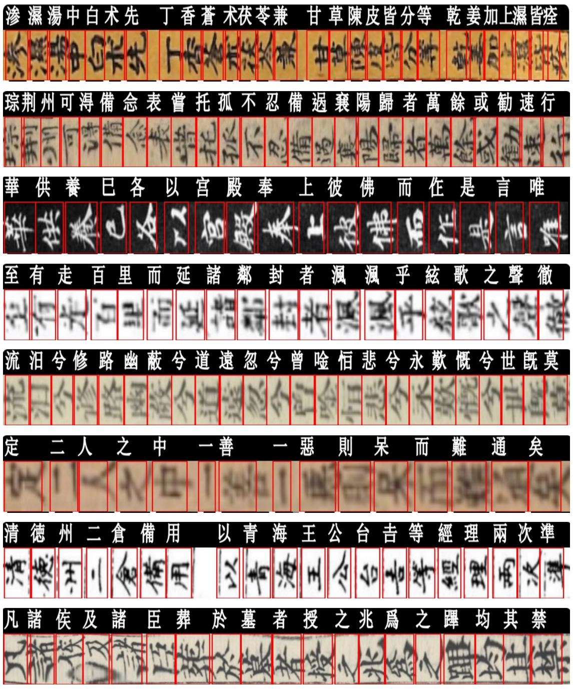

Figure 4: Visualization results on text line recognition.

References

[1] Cai Z and Vasconcelos N. Cascade R-CNN: Delving into high quality object detection. *Proceedings of the IEEE Conference on Computer Vision and Pattern Recognition* (2018) 6154–6162.

[2] Ma W, Zhang H, Jin L *et al.* Joint layout analysis, character detection and recognition for historical document digitization. *Proceedings of International Conference on Frontiers in Handwriting Recognition (ICFHR)* (2020) 31–36.

[3] Saini R, Dobson D, Morrey J *et al.* ICDAR 2019 historical document reading challenge on large structured Chinese family records. *Proceedings of International Conference on Document Analysis and Recognition (ICDAR)* (2019) 1499–1504.

[4] Liu Y, Zhang S, Jin L *et al.* Omnidirectional scene text detection with sequential-free box discretization. *Proceedings of the 28th International Joint Conference on Artificial Intelligence* (2019) 3052–3058.

[5] Wang W, Xie E, Song X *et al.* Efficient and accurate arbitrary-shaped text detection with pixel aggregation network. *Proceedings of the IEEE/CVF International Conference on Computer Vision* (2019) 8440–8449.

[6] Zhu Y, Chen J, Liang L *et al.* Fourier contour embedding for arbitrary-shaped text detection. *Proceedings of the IEEE/CVF Conference on Computer Vision and Pattern Recognition* (2021) 3123–3131.

[7] Liao M, Zou Z, Wan Z *et al.* Real-time scene text detection with differentiable binarization and adaptive scale fusion. *IEEE Transactions on Pattern Analysis and Machine Intelligence* 2022; **45**: 919–931.

[8] Shi B, Bai X and Yao C. An end-to-end trainable neural network for image-based sequence recognition and its application to scene text recognition. *IEEE Transactions on Pattern Analysis and Machine Intelligence* 2016; **39**: 2298–2304.

[9] Huang Y, Jin L and Peng D. Zero-shot chinese text recognition via matching class embedding. *Proceedings of International Conference on Document Analysis and Recognition (ICDAR)*, (Springer2021) 127–141.
